# Mapping implementation strategies of evidence-based interventions for three preselected phenomena in people with dementia—a scoping review

**DOI:** 10.1186/s43058-023-00486-4

**Published:** 2023-08-28

**Authors:** Mike Rommerskirch-Manietta, Christina Manietta, Daniel Purwins, Jana Isabelle Braunwarth, Tina Quasdorf, Martina Roes

**Affiliations:** 1 Deutsches Zentrum für Neurodegenerative Erkrankungen (DZNE), Site Witten, Witten, Germany; 2 Department of Nursing Science, Faculty of Health, Witten/Herdecke University, Witten, Germany; 3 School of Health Science, Institute of Nursing, ZHAW Zürich University of Applied Science, Winterthur, Switzerland

**Keywords:** Implementation science, ERIC, CFIR, Outcomes, Dementia

## Abstract

**Background:**

Caring for people with dementia is complex, and there are various evidence-based interventions. However, a gap exists between the available interventions and how to implement them. The objectives of our review are to identify implementation strategies, implementation outcomes, and influencing factors for the implementation of evidence-based interventions that focus on three preselected phenomena in people with dementia: (A) behavior that challenges supporting a person with dementia in long-term care, (B) delirium in acute care, and (C) postacute care needs.

**Methods:**

We conducted a scoping review according to the description of the Joanna Briggs Institute. We searched MEDLINE, CINAHL, and PsycINFO. For the data analysis, we conducted deductive content analysis. For this analysis, we used the Expert Recommendations for Implementation Change (ERIC), implementation outcomes according to Proctor and colleagues, and the Consolidated Framework for Implementation Research (CFIR).

**Results:**

We identified 362 (A), 544 (B), and 714 records (C) on the three phenomena and included 7 (A), 3 (B), and 3 (C) studies. Among the studies, nine reported on the implementation strategies they used. Clusters with the most reported strategies were *adapt and tailor to context* and train *and educate stakeholders*. We identified one study that tested the effectiveness of the applied implementation strategy, while ten studies reported implementation outcomes (mostly *fidelity*). Regarding factors that influence implementation, all identified studies reported between 1 and 19 factors. The most reported factors were *available resources* and the *adaptability* of the intervention. To address dementia-specific influencing factors, we enhanced the CFIR construct of *patient needs and resources* to include *family needs and resources*.

**Conclusions:**

We found a high degree of homogeneity across the different dementia phenomena, the evidence-based interventions, and the care settings in terms of the implementation strategies used, implementation outcomes measured, and influencing factors identified. However, it remains unclear to what extent implementation strategies themselves are evidence-based and which intervention strategy can be used by practitioners when either the implementation outcomes are not adjusted to the implementation strategy and/or the effects of implementation strategies are mostly unknown. Future research needs to focus on investigating the effectiveness of implementation strategies for evidence-based interventions for dementia care.

**Trial registration:**

The review protocol was prospectively published (Manietta et al., BMJ Open 11:e051611, 2021).

**Supplementary Information:**

The online version contains supplementary material available at 10.1186/s43058-023-00486-4.

Contributions to the literature

- ▪ To our knowledge, this study was the first to systematically identify implementation strategies, implementation outcomes, and influencing factors across preselected phenomena in people with dementia in different care settings.
- ▪ Established frameworks were used and enhanced for the analysis (dementia-specific adaptations of the CFIR) to advance the use of a consistent taxonomy in the field of implementation research in dementia care.
- ▪ The identified theory-guided implementation strategies and influencing factors can be used/considered to translate evidence-based knowledge into dementia care practice.
- ▪ Developing and testing discrete, multifaceted, and tailored implementation strategies seems necessary and will increase the impact of implementation studies, not only in dementia care research but also in other fields.

## Background

Healthcare for people with dementia appears to be more complex and challenging due to the symptoms of dementia, associated care needs, higher risks, and more frequent complications than for older people without dementia [1–4].

International studies have found that a high percentage of people with dementia in long-term care settings show behaviors that challenge healthcare professionals, such as agitation or aggression [5, 6]. This behavior is associated with an increased burden on healthcare professionals [7] and, in the setting of long-term care, increased prescribing of psychotropic drugs for people with dementia [8–10]. This, in turn, leads to decreased quality of life [11] and a possible increase in adverse effects such as risk of falls, an increase in medication that may lead to a sedated status, and, in the worst case, mortality [12, 13]. Furthermore, people with dementia are more likely to be hospitalized, have longer hospital stays, develop delirium that is more often undiagnosed, and experience a decline in their capacity to perform the activities of daily living [14–18]. As a result, the transition process (here, discharge from hospital to home or nursing home) and postacute care needs are more complex, challenging, and are associated with poorer outcomes than for older people without dementia [19, 20].

Internationally, an increasing number of psychosocial evidence-based interventions are focusing on these challenges and aimed at improving care outcomes for people with dementia [21–25]. Study results show that despite the increasing number of evidence-based interventions, patients receive only 30–40% of their care in line with the current scientific evidence, and in 20–25% of patients, there is a risk of harm in care [26].

Furthermore, healthcare professionals report that they implement research findings relatively seldomly in a structured and systematic way in their care practice [27]. This implementation gap has been researched thoroughly. For example, regarding the prescription and administration of psychotropic drugs to people with dementia in long-term care to reduce behaviors that challenge healthcare professionals. Although this has been shown to increase mortality since 2005 and there is poor evidence of effectiveness in improving symptoms [12], implementation and provision of evidence-based alternatives such as psychosocial interventions [28] do not appear to be used as a first approach [9, 29]. This is partly because implementing evidence-based interventions appears to be complex for healthcare staff, and there is often a lack of knowledge about how to implement interventions in a structured way [30–33].

## Implementation models, frameworks, and recommendations

To address this knowledge gap and further advance the implementation of, e.g., evidence-based interventions, various implementation models, frameworks, and recommendations for practitioners, researchers and other stakeholders exist. Among the best known are the Consolidated Framework for Implementation Research (CFIR) [34], the Expert Recommendations for Implementing Change (ERIC) [35, 36], and implementation outcomes according to Proctor, Silmere [37], which represent core concepts addressed by implementation science: facilitators and barriers to implementation [35], strategies to support implementation [32, 33], and implementation outcomes [34].

To evaluate the success of an implementation process, it is important to focus on the influencing factors for the implementation. Considering and identifying these factors can help to better select and design the implementation strategy up front [38], make appropriate adjustments during implementation, and gain a better understanding of what did or did not work and how and why after implementation has been completed. The CFIR provides a comprehensive description of these factors, which are divided into five major domains (intervention characteristics, outer setting, inner setting, characteristics of the individuals involved, and the process of implementation) [34].

The ERIC provides a comprehensive overview of 73 relevant implementation strategies that can be used individually or in combination by practitioners and researchers to implement interventions in care, for example [35, 36]. To assess whether an implementation has been successful and which implementation strategies are more effective, these strategies need to be tested and compared against predetermined implementation outcomes. Proctor, Silmere [37] have provided an overview of eight different implementation outcomes (acceptability, adoption, appropriateness, feasibility, fidelity, implementation cost, penetration, and sustainability), their level of analysis, theoretical basis, salience by implementation stage, and available measurements.

## Research questions

To our knowledge, there is no comprehensive, systematized evidence on implementation strategies, implementation outcomes and factors that influence the implementation of evidence-based interventions, which address the three phenomena that arise from the challenges in dementia care described above. Therefore, we developed the following three research questions:

- ▪ Which implementation strategies are promising for the implementation of evidence-based interventions for three preselected phenomena: (A) behavior that challenges supporting a person with dementia in long-term care, (B) delirium in acute care, and (C) postacute care needs?
- ▪ What are the effects of these implementation strategies on implementation outcomes?
- ▪ What are the factors that influence the implementation of evidence-based interventions?

## Methods

We described our methodological approach for the scoping review in our published review protocol [39], and according to Pieper, Ge [40], we reused the text of our review protocol for the methods sections in this publication and made changes in the method section where the process differed between the planned and conducted methodological approach. For reporting our scoping review, we use the Preferred Reporting Items for Systematic reviews and Meta-Analyses extension for Scoping Reviews (PRISMA-ScR) Checklist [41], as applicable (Supplementary Table 1). Additionally, we used the flow chart of the updated Preferred Reporting Items for Systematic Reviews and Meta-Analyses (PRISMA 2020) guidelines [42] to report the three literature searches (A, B, and C).

### Search strategies

To identify evidence-based interventions addressing the preselected phenomena (A, B, and C), two researchers (MR and TQ) conducted a narrative literature search in the MEDLINE (via PubMed), CINAHL and PsycINFO (via EBSCO) databases. We identified interventions that have been tested for feasibility and effectiveness and addressed our preselected phenomena. This led to the identification of these three key interventions: the Describe, Investigate, Create and Evaluate (DICE) approach for behavior that challenges supporting a person with dementia in long-term care [43], delirium management interventions (screening, assessment, monitoring, nonpharmacological interventions) [44], and the transitional care model (TCM) for the management of postacute care needs [45]. We used these interventions as starting points to develop our search string.

To develop a broad search string, we operationalized the interventions and their components into search terms. We also used other, broader terms for our identified interventions (e.g., person-centered care or transitional care) to avoid limiting ourselves to only those interventions identified up front. We supplemented these with search terms derived from our research questions (population, phenomena, implementation, setting). In addition, we used an initial search (MRM, JIB, CM and DP) in MEDLINE (via PubMed) and key publications to identify free search terms and indexing words. We clustered all of these search terms and indexing words according to the Population, Concept, and Context (PCC) mnemonic [46] and developed three different search strings (Supplemental Tables 2, 3, and 4). The search strings were developed by the researchers (A and B: MRM; C: CM), who have a professional background as nurses and have enhanced expertise in conducting reviews [47–52]. Furthermore, all three search strings were checked by all researchers (JIB, DP, TQ, MR) according to the Peer Review of Electronic Search Strategies (PRESS) guideline statements [53]. The search strings were first developed for MEDLINE (via PubMed) and were adapted for the other two databases (CINAHL and PsycINFO via EBSCO) according to the descriptions of RefHunter V.5.0 [54]. Search strategies for all three phenomena (A, B, and C) are reported in Supplementary Tables 2, 3, and 4. We searched MEDLINE (via PubMed), CINAHL, and PsycINFO (via EBSCO) between May and June 2021 and updated the search in June 2023. In addition, we conducted backward and forward citation tracking via reference lists and Google Scholar.

### Selection of evidence sources

In the first step, the abovementioned first reviewers of each review (MRM: A and B; CM: C) imported the identified records under three separate Covidence [55] licenses, and records for each search were checked automatically in Covidence for duplicates. In the second step, the titles and abstracts of each search were screened independently by two reviewers (A and B: MRM and JIB; C: CM and DP) against the inclusion and exclusion criteria (Table 1). Discrepancies in the voting were first discussed between reviewers, and if consensus could not be reached, they were discussed and resolved by all researchers (MRM, JIB, CM, DP, TQ, MR) in regular video meetings. Third, full-text screening was conducted by the same two reviewers independently (A and B: MRM and JIB; C: CM and DP), and discrepancies in the voting were discussed and resolved in the same manner as in the title and abstract screening.

**Table 1:** Inclusion and exclusion criteria [39]

**Criteria**	**Definition**
*Population*	▪ People with symptoms of dementia (with and without a dementia/an Alzheimer’s diagnosis) as the target population for the evidence-based interventions
*Concept of interest*	▪ Implementation of evidence-based interventions for the following phenomena: a) Behavior that challenges supporting a person with dementia b) Delirium c) Postacute care needs
*Context*	a) Long-term care b) Acute care c) Acute care
*Types of evidence sources*	▪ Any kind of study that describes or evaluates the implementation process of interventions (e.g., within the context of trials such as randomized controlled trials, hybrid design or daily practice)
*Other*	▪ Languages: German and English ▪ Year: no restrictions

### Data extraction

Our data extraction form was based on the template for scoping reviews developed by the Joanna Briggs Institute [46]. We considered the following aspects: *general information* (primary and additional publication, country, setting), *study design and methods* (aim, study design, methods), *participants* (sites and study population), and *intervention* (description of the implemented intervention, target population of the intervention). Data extraction for each search was performed independently by two researchers (A and B: MRM and JIB; C: CM and DP). Deviations in the extraction were discussed first between the two researchers and, if a consensus could not be reached, with all researchers (MRM, JIB, CM, DP, TQ, MR) in regular video meetings.

### Analysis of the evidence

For the analysis of implementation strategies, implementation outcomes, and factors influencing implementation reported in the identified studies, we used a deductive content analysis approach [56]. For this, we derived the categories from ERIC [35, 36, 57] to analyze the implementation strategies used in the identified studies. Because implementation outcomes were often not explicitly stated and reported in the included studies, we used the outcomes described by Proctor, Silmere [37] to identify and analyze implementation outcomes in the included studies. Additionally, we used the five domains of the Consolidated Framework for Implementation Research (CFIR) and their constructs [34] to analyze the reported influencing factors.

For the coding process of implementation strategies and outcomes, as well as influencing factors, the results of each search were independently coded by two reviewers (A and B: MRM and JIB; C: CM and DP). Afterward, the results for each coding were compared, and discrepancies were discussed in the two groups (A and B; C). Codes that could not be clearly assigned to one category were discussed with all researchers (MRM, JIB, CM, DP, TQ, MR) in a virtual meeting. After the coding process, all codings were peer checked by one of two researchers (TQ or MR) to ensure trustworthiness [58].

### Presentation of results

For the presentation of our scoping review results, we mapped the implementation strategies and outcomes, as well as the influencing factors, in the form of 3 tables with tick boxes. In addition, we report further detailed information about the various identified in a descriptive way.

## Results

**Fig. 1:**
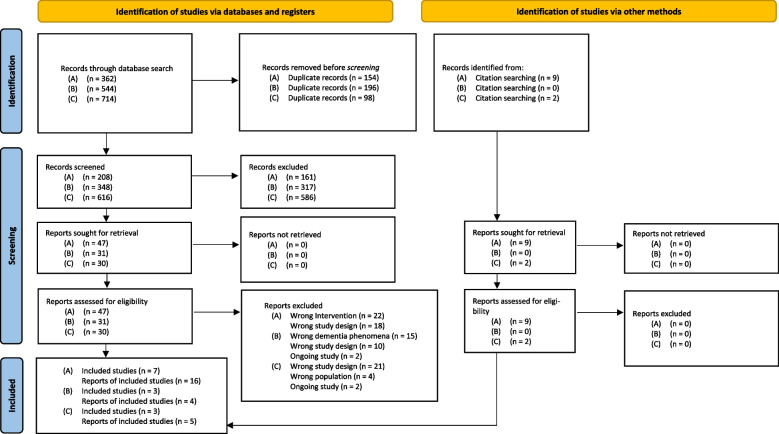
PRISMA 2020 flow diagram [42] demonstrating the identification, screening, and eligibility assessments of records preceding scoping review inclusion

Through our electronic database searches, we identified a total of 362 (A: behavior that challenges supporting a person with dementia in long-term care), 544 (B: delirium in acute care), and 714 records (C: postacute care needs). After removing duplicates, we screened 208 (A), 348 (B), and 616 (C) records against our inclusion and exclusion criteria. Ultimately, we included 7 [59–65] (A), 3 [66–69] (B), and 3 [70–72] (C) studies. In addition, we identified 9 [73–80] (A) and 2 [81, 82] (C) corresponding reports through our backward and forward citation tracking of the studies that were included in the review (Fig. 1).

### Study characteristics

Most of the studies were from Australia (*n* = 6) [60, 61, 67, 69, 71, 72] or the USA (*n* = 6) [59, 62, 64–66, 70], and there was one study from the UK [63]. The study designs of the included primary studies included implementation studies (*n* = 4) [65, 67, 69, 72], projects (*n* = 3) [59, 62, 64], process evaluations (*n* = 2) [61, 71], pilot/feasibility studies (*n* = 2) [63, 66], pre/post design (*n* = 1) [60], and one qualitative study (*n* = 1) [70]. The number of participating healthcare professionals (*n* = 1079) was reported in ten studies [61–67, 69–71]. In addition, ten studies reported the number of participating people with dementia and/or patients and their relatives (*n* = 1435) [61, 63–67, 69–72]. Detailed information about the study characteristics (e.g., implemented interventions) of all included studies is provided in Table 2.

**Table 2:** Study characteristics

**General information**	**Study design and methods**	**Participants**	**Intervention/survey name/interview guideline**
**(A) Behavior that challenges supporting a person with dementia in long-term care**			
**Primary publication:** [60] **Additional publication:** no information **Country:** Australia **Setting:** residential aged care facilities	**Aim:** to determine the effectiveness and acceptability of the concept mapping approach **Study design:** quasi-experimental, pre/post testing design **▪ Methods:** staff questionnaires, care plan audits, concept mapping audits	**Sites** **▪** Residential aged care facilities (*n* = 11) **Study population** **▪** Nursing staff^a^	**Concept mapping** Case conferences with: **▪** Two formal assessments and perspectives of the team were brought in the mapping process **▪** The content of the concept map needed to be accurate, fact- or evidence-based and linked to existing concepts **▪** The concept map needed to be validated against what was known about the person by family and staff **▪** The concept map guided care plan development/evaluation and helped to identify potential risks for staff and clients **Target population of the intervention:** **▪** Nursing staff (registered and enrolled nurses, personal care workers)
**Primary publication:** [59] **Additional publication:** [73] **Country:** USA **Setting:** nursing home	**Aim:** to implement the Evidence Integration Triangle for Management of Behavioral Psychological Symptoms of Dementia (EIT-4-BPSD) **Study design:** quality improvement project **▪ Methods:** routine data (residents), tests, observations (nursing staff), assessments (facility), document analysis (nursing staff/facility)	**Sites** **▪** Nursing home (*n* = 1) **Study population** **▪** Nursing staff^a^ **▪** Residents^a^	**EIT-4-BPSD** Consists of four steps: 1. Step: Assessing the environment and policies 2. Step: Educating staff 3. Step: Establishing person-centered care plans 4. Step: Mentoring and motivating staff **Target population of the intervention:** **▪** Facility **▪** Nursing staff **▪** Residents, who were 55 years and older with at least one behavior that challenges supporting a person with dementia within the past month
**Primary publication:** [61] **Additional publications:** [74, 75] **Country:** Australia **Setting:** residential dementia care home	**Aim:** to understand the inconsistent results of the main study and investigation of factors that enabled and inhibited the implementation of the intervention **Study design:** process evaluation of a randomized controlled trial **▪ Methods:** interviews with care managers, nurses, and care staff, surveys with family members, staff reports, field notes, care plans	**Sites** **▪** Residential dementia care homes (*n* = 38) **Study population** **▪** Care managers (*n* = 29) **▪** Nurses and care staff (*n* = 70) **▪** Family members (*n* = 73)	**Person-centered care (PCC) and person-centered dementia environment (PCE)** PCC consists of: **▪** Educational sessions for the staff with a focus on paying attention to residents’ feelings when agitated, person-centered interactions, and person-centered care planning to meet psychosocial needs of the residents **▪** On-site supervision **▪** Telephone support PCE consisted of: **▪** Environmental audit **▪** Audit results regarding safety, accessibility, utility, colors, objects, and familiarity of outdoor and social space were considered, and facilities were modified **Target population of the intervention:** **▪** Nursing and care staff and facilities **▪** Indirect people with dementia aged ≥ 60 years
**Primary publication:** [62] **Additional publication:** no information **Country:** USA **Setting:** long-term care facility	**Aim:** to improve the skills of care staff for the care of people with dementia experiencing behavior that challenges supporting a person with dementia **Study design:** educational project **▪ Methods:** questionnaire	**Sites** **▪** Long-term care facility (*n* = 1) **Study population** First educational session: **▪** Staff from all departments (*n* = 165) Second to fifths educational session: **▪** Staff (*n* = 70)	**Educational program** The intervention consisted of five 30–45 min educational sessions: **▪** Introduction to dementia and the STAR-VA program **▪** (Non)verbal communication with people with/without dementia living in a long-term care facility setting **▪** Presentation and explanation of the DICE approach for behavior that challenges supporting a person with dementia **▪** Summary **Target population of the intervention:** **▪** Staff (registered nurses, licensed practice nurses, certified nursing assistants, activity staff, unit secretary, social workers, dietitians, nurse practitioners, housekeepers, dietary staff, charge nurses, unit managers, MDS coordinators and MDS supervisors)
**Primary publication:** [63] **Additional publication:** [76, 80] **Country:** UK **Setting:** nursing homes	**Aim:** to determine the feasibility of the implementation and effectiveness of a dual-purpose pharmacy-health psychology intervention **Study design:** open label, mixed method, feasibility study **▪ Methods:** interviews, reflective comments via interviews, questionnaire, medication records	**Sites** **▪** Nursing homes (*n* = 5) **Study population** **▪** Nursing home staff (*n* = 142) **▪** General healthcare staff (*n* = 22) **▪** People with dementia (*n* = 108)	**Dual-focused medication review-behavioral change intervention** Workshop: **▪** For nursing home staff, three educational workshops with a focus on person-centered care and the understanding that unmet needs could cause behavior that challenges supporting a person with dementia were conducted **▪** Aim of the workshop: to provide staff with skills for the investigation of unmet needs, knowing the person, individualized interventions to meet person needs, understanding that engaging in behavior that challenges supporting a person with dementia is not to be understood as engaging in bad behavior or being a bad person **▪** The training was conducted along the VIPS (Valuing, Individualized, Perspective, and Social) model and included educational elements regarding medication to manage behavior that challenges supporting a person with dementia, guidelines to reduce medication and favor psychosocial interventions and videos, demonstration of person-centered practices, and information about the abovementioned topics; the importance of self-care and good communication among care staff was discussed **▪** Primary healthcare staff received a modified version of the education workshops Medication review: **▪** Medication review was provided by two experienced clinical pharmacists **▪** A therapeutic alliance was formed between the clinical pharmacist, the person with dementia and their personal relative **▪** Information about the medications and adverse effects were collected **▪** Medication plans were reviewed, with a focus on medications for behavior that challenges supporting a person with dementia, and all other medications **▪** Information and recommendations about the review were provided to the general practitioner in writing and by telephone **Target population of the intervention:** **▪** Nursing home staff **▪** General healthcare staff [general practitioner (trainees), practice nurses, practice-based pharmacist] **▪** People with dementia who received medication to treat behavior that challenges supporting a person with dementia
**Primary publication:** [64] **Additional publication:** no information **Country:** USA **Setting:** nursing homes	**Aim:** to change the culture of care in nursing homes, establishing a person-centered model, and reducing the treatment of behavior that challenges supporting a person with dementia with antipsychotic medications **Study design:** quality improvement initiative **▪ Methods:** survey, chart reviews, questionnaire	**Sites** **▪** One dementia care unit of a nursing home **Study population** **▪** Administrative team (facility administrator, medical director, director of nursing, human resources, clinical nurse educator, and the scheduler) **▪** Direct care team [doctors *n* = 3, (hospice) nurse practitioner *n* = 2, unit nurse manager (*n* = 1), activity director (*n* = 1 with two assistants, certified nurse assistants *n* = 18 and licensed practical nurses *n* = 10 on the unit, clinical nurse educator, wound care nurse, and social worker] **▪** Residents (*n* = 39) **▪** Families of the residents^a^	**No specific name** The interventions targeted residents and staff Residents: **▪** Waking up time of the residents postponed by two hours **▪** Increase in guided activities during the day **▪** Bathing could be postponed to the evening **▪** Adjustment of the time for the administration of the medication Staff: **▪** Establishing a resident-guided schedule for morning caregiving, medication administration and breakfasts routine **▪** Increase staff to resident ratio **▪** Workshops to educate on culture change and risk of antipsychotics **Target population of the intervention:** **▪** All residents of the dementia care unit **▪** Administrative team **▪** Direct care team
**Primary publication:** [65] **Additional publications:** [77–79, 83] **Country:** USA **Setting:** nursing homes	**Aim:** to implement, test, and evaluate the implementation strategy of the Evidence Integration Triangle for Management of Behavioral Psychological Symptoms of Dementia (EIT-4-BPSD) **Study design:** pilot study (single-group repeated measures study), testing implementation strategy and process evaluation **▪ Methods:** focus groups, medical records, assessments, care plans, checklist, test, observations	**Sites** Pilot study: **▪** Nursing homes (*n* = 2) Implementation study: **▪** Nursing homes (*n* = 55) **Study population** Pilot study: **▪** Residents (*n* = 21) Implementation study and process evaluation: **▪** Nursing home staff [(*n* = 93), administrator *n* = 5, director of nursing *n* = 17, staff nurse *n* = 18, nursing assistant *n* = 6, social worker/social service *n* = 12, recreation therapist/activity staff *n* = 17, others *n* = 10] **▪** Residents (*n* = 553)	**EIT-4-BPSD** Consists of an initial brainstorming and four steps: **▪** Initial 4-h brainstorming session with site champion and stakeholder team 1. Step: Assessing the environment and policies 2. Step: Educating staff 3. Step: Establishing person-centered care plans 4. Step: Mentoring and motivating staff **Control group (education only)** **▪** Step 2: Educating staff **Target population of the intervention:** **▪** Facility **▪** Nursing home staff **▪** Residents, who were 55 years and older with at least one behavior that challenges supporting a person with dementia within the past month
**(B) Delirium in acute care**			
**Primary publication:** [66] **Additional publication:** no information **Country:** USA **Setting:** acute care hospital	**Aim:** to investigate the feasibility of the computerized decision support component of a multicomponent intervention **Study design:** prospective, cohort, pilot study **▪ Methods:** instruments, interview, surveys, telephone calls, field notes, hospital information services staff information, feedbacks	**Sites** **▪** One adult medical-surgical unit in an acute care hospital **Study population** **▪** Registered nurses (*n* = 55) **▪** Licensed practice nurses (*n* = 19) **▪** Patients with dementia (*n* = 15)	**Early Nurse Detection of Delirium Superimposed on Dementia** The intervention consisted of four components: **▪** Education for nurses with a focus on detection and management of delirium superimposed on dementia **▪** Delirium decision support screens integrated in a computerized decision support system **▪** A champion to support the implementation per unit **▪** A feedback system to individual nurses on each intervention with the aim to further facilitate assessment and management of delirium superimposed on dementia **Target population of the intervention:** **▪** Registered and licensed practice nurses **▪** Patients with dementia aged ≥ 65 years
**Primary publication:** [67] **Additional publication:** no information **Country:** Australia **Setting:** acute hospitals	**Aim:** to evaluate the implementation of a model of care **Study design:** pre/post implementation study **▪ Methods:** medical record, audits, surveys, interviews	**Sites** **▪** Acute hospitals (*n* = 6) **Study population** **▪** Nursing staff (pre *n* = 432; post *n* = 283) **▪** Patients (pre *n* = 347; post *n* = 396)	**Confused Hospitalized Older Persons (CHOPs)** The intervention consists of seven principles: **▪** Cognitive screening **▪** Identification and prevention strategies for delirium **▪** Assessment of older people with confusion **▪** Communication to support person-centered care **▪** Staff education related to caring for older people with confusion **▪** Supportive care environments for older people with confusion **Target population of the intervention:** **▪** Nursing staff **▪** Direct and indirect patients aged ≥ 65 years
**Primary publication:** [69] **Additional publication:** [68] **Country:** Australia **Setting:** acute hospital	**Aim:** to implement a multifaceted practice change intervention to enhance the capacity of the nursing staff to provide quality care **Study design:** implementation study **▪ Methods:** observation, audits, assessments	**Sites** **▪** Wards (*n* = 6) in one acute hospital **Study population** **▪** Registered nurses (*n* = 34) **▪** Patients (*n* = 181)	**Cognition Champions (CogChamps)** The intervention consisted of three steps: **▪** 2 education workshops for CogChamps **▪** development of an individualized action plan by CogChamps **▪** Implementation of actions plans by CogChamps **Target population of the intervention:** **▪** Registered nurses with two or more years of clinical experience **▪** Indirect patients aged ≥ 65 years
**(C) Postacute care needs**			
**Primary publication:** [72] **Additional publication:** [81] **Country:** Australia **Setting:** hospital	**Aim:** to improve hospital discharge processes for older people with dementia **Study design:** effectiveness-implementation hybrid design **Methods:** conducting three phases: **▪** Phase 1 organizational readiness: analysis of policy and practice documents related to discharge, interviews, workshops summaries **▪** Phase 2 development of a discharge intervention: survey, meeting minutes, workshop summaries **▪** Phase 3 implementation and evaluation: survey, interviews, meeting minutes, administrative data, study notes	**Sites** **▪** 4 wards (with 24 to 32 beds) in 3 hospitals **Study population** **▪** Phase 1: clinical staff and families of patients with dementia in participating wards^a^ **▪** Phase 2: Queensland health clinical staff^a^, researchers^a^, consumers^a^, representatives (state wide clinical networks for older persons health and dementia)^a^ **▪** Phase 3: clinical staff and families of patients with dementia in participating wards^a^, local hospital staff^a^ and stakeholders^a^ in each hospital, patients with dementia (*n* = 44)	**Partnering for Discharge** The intervention consists of four elements: **▪** My Hospital Guide: a person-centered guide with information for the people with dementia and their relatives about the hospital stay and offers **▪** My Journal: a document with information and questions regarding care and discharge, held by the patient or family **▪** This is me: a document that records the person’s background, preferences, and interests **▪** Family meeting within 72 h of admission **Target population of the intervention:** **▪** patients aged ≥ 65 years diagnosed with dementia (primary or additional diagnosis) **▪** family members of patients with diagnosed dementia
**Primary publication:** [71] **Additional publication:** no information **Country:** Australia **Setting:** residential care facility	**Aim:** to evaluate the implementation and effectiveness of the TC CAMP **Study design:** evaluation (process and outcome) **Methods:** individual and focus group interviews, file audits (medical records)	**Sites** **▪** 6 restorative care places in a dementia unit in one residential facility **Study population** **▪** TC Camp staff (*n* = 7) **▪** Health service staff (*n* = 7) **▪** Representatives of the facility to which clients were discharged (*n* = 3) **▪** Clients with dementia (*n* = 11) **▪** Family members/carers (*n* = 7)	**Transition Care Cognitive Assessment and Management Pilot (TC CAMP)** TC Camp is based on a person-centered approach as a goal-oriented and time-limited healthcare service for people with dementia who were discharged from the hospital TC Camp includes the following components: **▪** Clinical nurse consultant (CNC) [Role of the CNC: Case management including family meetings, cognitive assessment, behavior management, discharge planning, and staff education) **▪** Geriatrician **▪** Occupational therapist **▪** Other health professions if required **▪** Person centered tool “Key to me” **▪** Individualized care/behavioral and discharge plan **Target population of the intervention:** **▪** Patients aged ≥ 65 years with cognitive impairment (MMSE ≤ 24)
**Primary publication:** [70] **Additional publication:** [82] **Country:** USA **Setting:** hospital, postacute setting	**Aim:** to examine barriers and facilitators for implementing a transitional care intervention for cognitively impaired older adults and their caregivers **Study design:** exploratory qualitative design **Methods:** case summaries (of each patient caregiver dyad), case conference field notes	**Sites** **▪** Hospitals^a^ **Study population** **▪** Advanced practice nurses (APNs) (*n* = 3) **▪** Caregivers of patients with dementia (*n* = 16) **▪** Patients with dementia (*n* = 15)	**Transitional Care Model (TCM)** The TCM is based on the APN role and includes hospital, home, and discharge components General: **▪** The APN develops goals with the patients and their caregivers, identifies teaching and learning needs, and considers other needs and issues impacting the care **▪** Scheduled visits from the APN: first visit within 24 h after admission, daily visits during the hospitalization, first visit within 24 h after discharge, weekly visits during the first month after discharge plus telephone contact, if needed, at least once per week when no home visit is scheduled, at least semimonthly visits during the intervention period Hospital components: **▪** Relationship building with the patients/carers, implementing prevention strategies (e.g., effects of cognitive impairment), developing and implementing of individualized care plans Home component: **▪** Starts immediately after the discharge, availability of the APN 7 days per week (8 a. m.–8 p. m.), development of an individual emergency care plan (for the time when the APN is unavailable), support and structuring of the first visit to the primary care or specialist healthcare provider Discharge: **▪** APNs use their clinical assessment skills to determine the time of the intervention, the termination of the discharge is guided by medical stability, patient and caregiver goals, and the skills of the caregiver to identify early symptoms that require intervention and strategies to prevent poor outcomes **Target population of the intervention:** **▪** Patients aged ≥ 65 years with cognitive impairment (six-item screen ≤ 4) living at home **▪** Caregiver

^a^(*n*) not reported

### Identified implementation strategies

In the included studies that reported implementation strategies, we were able to identify between 4 and 21 ERIC strategies per study (Table 3). The two clusters with the most reported implementation strategies were *adapt and tailor to context* (3 of 4, 75% reported on *tailor strategies*, *promote adaptability*, and *use data experts*) and *train and educate stakeholders* (8 of 11, 73% reported on *conduct ongoing training*, *provide ongoing consultation*, *develop educational materials*, *make training dynamic*, *distribute educational materials*, *use train the trainer strategies*, *conduct educational meetings*, and *work with educational institutions*) (Table 3).

**Table 3:** Implementation strategies across the different phenomena in dementia care

**Clusters and relevant implementation strategies of the ERIC**		[60]	[59, 73]	[61, 74, 75]	[62]	[63, 76, 80]	[64]	[65, 77–79, 83]		[66]	[67]	[68, 69]		[72, 81]	[71]	[70, 82]	***N***
***Use evaluative and iterative strategies***	**(A) Behavior that challenges supporting a person with dementia in long-term care**								**(B) Delirium in acute care**				**(C) Postacute care needs**				
Assess for readiness and identify barriers and facilitators							***x***	***x***		***x***	***x***	***x***		***x***			**6**
Audit and provide feedback			***x***	***x***	***x***			***x***			***x***	***x***		***x***			**7**
Develop and organize quality monitoring											***x***	***x***					**2**
Develop a formal implementation blueprint			***x***		***x***		***x***	***x***			***x***	***x***		***x***			**7**
Stage implementation scale up								***x***			***x***						**2**
***Provide interactive assistance***																	
Facilitation								***x***				***x***					**2**
***Adapt and tailor to context***																	
Tailor strategies					***x***			***x***				***x***		***x***			**4**
Promote adaptability					***x***		***x***	***x***						***x***			**4**
Use data experts											***x***						**1**
***Develop stakeholder interrelationships***																	
Identify and prepare champions			***x***	***x***			***x***	***x***			***x***	***x***		***x***			**7**
Organize clinician implementation team meetings								***x***			***x***			***x***			**3**
Recruit, designate, and train for leadership				***x***													**1**
Inform local opinion leaders												***x***					**1**
Build a coalition								***x***			***x***	***x***					**3**
Obtain formal commitments								***x***				***x***					**2**
Capture and share local knowledge											***x***						**1**
Use advisory boards and workgroups			***x***		***x***		***x***	***x***			***x***	***x***		***x***			**7**
Use an implementation advisor			***x***	***x***				***x***			***x***	***x***					**5**
***Train and educate stakeholders***																	
Conduct ongoing training				***x***				***x***				***x***					**3**
Provide ongoing consultation							***x***	***x***			***x***						**3**
Develop educational materials			***x***		***x***						***x***	***x***					**4**
Make training dynamic												***x***					**1**
Distribute educational materials								***x***		***x***	***x***						**3**
Use train-the-trainer strategies			***x***	***x***													**2**
Conduct educational meetings							***x***	***x***				***x***		***x***			**4**
Work with educational institutions											***x***						**1**
***Support clinicians***																	
Facilitate relay of clinical data to providers											***x***						**1**
Remind clinicians												***x***					**1**
Create new clinical teams							***x***				***x***						**2**
***Engage consumers***																	
Involve patients/consumers and family members							***x***					***x***		***x***			**3**
Intervene with patients/consumers to enhance uptake and adherence										***x***							**1**
Use mass media												***x***					**1**
***Utilize financial strategies***																	
Fund and contract for the clinical innovation											***x***						**1**
Access new funding					***x***						***x***	***x***					**3**
***Change infrastructure***																	
Change physical structure and equipment										***x***	***x***	***x***					**3**

We identified the most common implementation strategies in two other ERIC clusters. For the cluster *develop stakeholder interrelationships*, we identified the following implementation strategies: *identify and prepare champions* (*n* = 7) [59, 61, 64, 67–69, 72, 78, 79, 81], *use advisory boards and workgroups* (*n* = 7) [59, 62, 64, 67–69, 72, 78, 79, 81], and *use an implementation advisor* (*n* = 5) [59, 61, 67, 69, 75, 78, 79]. In the cluster *use evaluative and iterative strategies*, the following implementation strategies were identified: *audit and provide feedback* (*n* = 7) [59, 61, 62, 67–69, 72, 75, 78, 79, 81], *develop a formal implementation blueprint* (*n* = 7) [59, 62, 64, 67–69, 72, 78, 79, 81], and *assess readiness* (*n* = 6) [64, 66–69, 72, 78, 79, 81].

We were not able to identify 38 of the 73 ERIC implementation strategies. Most implementation strategies were not reported in these clusters: *change infrastructure* (7 of 8, 88% did not report on *mandate change*, *change record system*, *create or change credentialing and/or licensure standards*, *change service sites*, *change accreditation or membership requirements*, *start a dissemination organization*, or *change liability laws*), *utilize financial strategies* (7 of 9, 78% did not report on *place innovation on fee for service lists/formularies*, *alter incentive/allowance structures*, *make billing easier*, *alter patient/consumer fees*, *use other payment schemes*, *develop disincentives*, or *use capitated payments*), and *provide interactive assistance* (3 of 4, 75% did not report on *provide local technical assistance*, *provide clinical supervision*, or *centralize technical assistance)* (Table 3).

To gain deeper insight into the coding of the implementation strategies, we present examples in Table 4.

**Table 4:** Examples of codings for the most common clusters and implementation strategies

**Most common descripted clusters and implementation strategies**	**Example of coding**
***Adapt and tailor to context***	
Tailor strategies	*“EIT allows for differences between communities and encourages tailoring of the implementation process, in contrast to an explanatory trial in which strict adherence to the intervention protocol is maintained” *[78]
Promote adaptability	*“The usual training for the STAR-VA program requires two half-day sessions and then four individualized sessions. This would not be a viable plan at the project site. Five monthly sessions were then planned for 30 to 40 min in length, to fit into the workflow of the day.” *[62]
Use data experts	*“Completed hard copies were entered into SurveyMonkey™ by ACI staff” *[67]
***Train and educate stakeholders***	
Conduct ongoing training	*“Working together, these individuals enact the triad of components of EIT-4-BPSD, which include: (1) participatory implementation *via* a combination of in-person monthly meetings, weekly emails, and phone interactions between stakeholders and a research facilitator as they develop community goals and work toward achieving those goals…” *[78]
Provide ongoing consultation	*“Fortnightly teleconferences with the site clinical leads were facilitated by the CHOPs project officer. These provided regular mentoring support and the opportunity for clinical leads to report on their progress and share their experiences and solutions throughout the implementation” *[67]
Develop educational materials	*“The DNP student provided resource binders containing additional resources on BPSD from the nursing home toolkit website. Binders were placed at each nursing station.” *[59]
Make training dynamic	*“…conducting education sessions, providing bedside teaching and role-modeling best practices, sourcing resources and maintaining records” *[69]
Distribute educational materials	*“The nurses were also given pocket cards for sleep hygiene, the MMSE, and the CAM.” *[66]
Use train the trainer strategies	*“For the PCC intervention, we employed a train-the-trainer-staff coaching model and engaged staff champions to cocreate and disseminate PCC knowledge among work teams” *[61]
Conduct educational meetings	*“The Facilitator CogChamps undertook a very active role in working with the other CogChamps to assist them in making progress with their action plans. They provided direct support by conducting education sessions, providing bedside teaching and role-modeling best practices, sourcing resources and maintaining records” *[69]
Work with educational institutions	*“…workshop sessions and facilitated e-learning through the NSW Dementia Competency and Training Network” *[67]
***Develop stakeholder interrelationships***	
Identify and prepare champions	*“For the PCC intervention, we employed a train-the-trainer-staff coaching model and engaged staff champions to cocreate and disseminate PCC knowledge among work teams”*[61]
Use advisory boards and workgroups	*“The purpose of the committee is to provide support and guidance regarding the project’s implementation” *[68]
Use an implementation advisor	*“Evidence Integration Triangle for Behavioral and Psychological Symptoms of Dementia was implemented by the research nurse facilitator working with the internal champion and stakeholders using the 4-step approach…” *[79]
***Use evaluative and iterative strategies***	
Audit and provide feedback	*“Members of the research team assisted the Cog-Champs in implementing their action plans by meeting with one or more CogChamp(s) from each ward weekly (face to face and email) to assess progress, provide feedback, and support them over the five-month implementation phase” *[69]
Develop a formal implementation blueprint	*“…a project implementation plan written…” *[67]
Assess for readiness	*“In phase 1, organizational readiness was assessed,…” *[72]

### Effectiveness of the implementation strategies and outcomes

Only one study tested the effectiveness of the applied implementation strategy [65]: the effectiveness of the EIT-4-BPSD versus education only. In this study, implementation outcomes related to adoption, fidelity, penetration, and sustainability were reported. The effects of the implementation outcome sustainability were compared between both groups (intervention and control). In both groups, a slight increase in the policies and environment in terms of promoting person-centered care was observed. No change was noted in the person-centered design of care plans in either group. Related to other implementation outcomes (adoption, fidelity, and penetration), no results were reported for either group [65].

Of the remaining 12 studies that did not evaluate the effectiveness of their implementation strategy, ten reported implementation outcomes [59–63, 66, 67, 69, 71, 72]. Here, the outcomes fidelity (*n* = 10), acceptability (*n* = 5), adoption (*n* = 4), and penetration (*n* = 4) were reported most frequently (Table 5).

**Table 5:** Reported implementation outcomes for the included phenomena in dementia care

**Implementation outcomes according to Proctor, Silmere **[37]	**Acceptability**	**Adoption**	**Appropriateness**	**Feasibility**	**Fidelity**	**Implementation Cost**	**Penetration**	**Sustainability**
**(A) Behavior that challenges supporting a person with dementia in long-term care**								
[60]	***x***				***x***			
[59, 73]					***x***			***x***
[61, 74, 75]					***x***	***x***		
[62]					***x***			
[63, 76, 80]	***x***	***x***			***x***	***x***	***x***	
[64]								
[65, 77–79, 83]		***x***			***x***		***x***	***x***
**(B) Delirium in acute care**								
[66]	***x***	***x***		***x***	***x***		***x***	
[67]			***x***		***x***		***x***	
[68, 69]		***x***	***x***		***x***			
**(C) Postacute care needs**								
[72, 81]	***x***	***x***			***x***		***x***	
[71]	***x***		***x***		***x***			
[70, 82]								
***N* = **	**5**	**5**	**3**	**1**	**11**	**2**	**5**	**2**

### Identified influencing factors

We identified 28 of the 37 constructs of the CFIR in the included studies (Table 6). In the following, we describe the two most frequently mentioned constructs of each CFIR domain across the different phenomena in dementia care (a, b, and c). Due to the different structuring of the domain *inner setting*, the most frequent subcodes of the constructs *implementation climate* and *readiness for implementation* were also listed (Table 6).

**Table 6:** Influencing factors for the included phenomena in dementia care

**Relevant domains and** constructs** of the CFIR**		[60]	[59, 73]	[61, 74, 75]	[62]	[63, 76, 80]	[64]	[65, 77–79, 83]		[66]	[67]	[68, 69]		[72, 81]	[71]	[70, 82]	***N***
***Intervention characteristics—definition according to CFIRGuide*** [84]	**(A) Behavior that challenges supporting a person with dementia in long-term care**								**(B) Delirium in acute care**				**(C) Postacute care needs**				
Intervention source—p*erception of key stakeholders about whether the intervention is externally or internally developed*														***x***			***1***
Evidence strength and quality—*stakeholders’ perceptions of the quality and validity of evidence supporting the belief that the intervention will have the desired outcomes*		***x***				***x***	***x***	***x***							***x***	***x***	***6***
Adaptability—*the degree to which an intervention can be adapted, tailored, refined, or reinvented to meet local needs*				***x***	***x***		***x***	***x***		***x***		***x***		***x***	***x***	***x***	***8***
Complexity—*perceived difficulty of the intervention, reflected by duration, scope, radicalness, disruptiveness, centrality, and intricacy and number of steps required to implement*											***x***			***x***			***2***
***Outer setting—definition according to CFIRGuide*** [84]																	
Patient needs and resources/needs and resources of the family—*the extent to which patient needs, as well as barriers and facilitators to meet those needs, are accurately known and prioritized by the organization*				***x***			***x***	***x***								***x***	***4***
Cosmopolitanism—*the degree to which an organization is networked with external organizations*											***x***				***x***	***x***	***3***
External policy and incentives—*a broad construct that includes external strategies to spread interventions, including policy and regulations (governmental or other central entity), external mandates, recommendations and guidelines, pay-for-performance, collaboratives, and public or benchmark reporting*							***x***	***x***									***2***
***Inner setting—Definition according to CFIRGuide*** [84]																	
Structural characteristics—*the social architecture, age, maturity, and size of an organization*			***x***					***x***			***x***	***x***		***x***			***5***
Networks and communications—*the nature and quality of webs of social networks and the nature and quality of formal and informal communications within an organization*					***x***									***x***	***x***		***3***
Culture—*norms, values, and basic assumptions of a given organization*				***x***			***x***	***x***									***3***
*Implementation climate—Definition according to CFIRGuide* [84]																	
*Learning climate*—*a climate in which: a) leaders express their own fallibility and need for team members’ assistance and input; b) team members feel that they are essential, valued, and knowledgeable partners in the change process; c) individuals feel psychologically safe to try new methods; and d) there is sufficient time and space for reflective thinking and evaluation*				***x***				***x***						***x***		***x***	**4**
*Goals and feedback*—*the degree to which goals are clearly communicated, acted upon, and fed back to staff, and alignment of that feedback with goals*				***x***										***x***			**2**
*Organizational incentives and rewards*—*extrinsic incentives such as goal-sharing awards, performance reviews, promotions, and raises in salary, and less tangible incentives such as increased stature or respect*				***x***				***x***									**2**
*Relative priority*—*individuals’ shared perception of the importance of the implementation within the organization*						***x***		***x***			***x***						**3**
*Compatibility*—*the degree of tangible fit between meaning and values attached to the intervention by involved individuals, how those align with individuals’ own norms, values, and perceived risks and needs, and how the intervention fits with existing workflows and systems*								***x***			***x***				***x***		**3**
*Tension for change*—*the degree to which stakeholders perceive the current situation as intolerable or needing change*				***x***	***x***			***x***									**3**
*Readiness for implementation—definition according to CFIRGuide* [84]																	
*Access to knowledge and information*—*ease of access to digestible information and knowledge about the intervention and how to incorporate it into work tasks*				***x***	***x***		***x***				***x***				***x***		***5***
*Available resources*—*the level of resources dedicated for implementation and on-going operations, including money, training, education, physical space, and time*		***x***	***x***	***x***	***x***	***x***		***x***			***x***	***x***		***x***	***x***		***10***
*Leadership engagement*—*commitment, involvement, and accountability of leaders and managers involved in the implementation*				***x***	***x***			***x***			***x***						***4***
***Characteristics of individuals—definition according to CFIRGuide*** [84]																	
Knowledge and beliefs about the intervention—individuals’ attitudes toward and value placed on the intervention as well as familiarity with facts, truths, and principles related to the intervention		***x***		***x***		***x***	***x***	***x***						***x***	***x***		***7***
Individual stage of change—characterization of the phase an individual is in, as he or she progresses toward skilled, enthusiastic, and sustained use of the intervention		***x***		***x***													***2***
Other personal attributes—a broad construct to include other personal traits such as tolerance of ambiguity, intellectual ability, motivation, values, competence, capacity, and learning style				***x***	***x***			***x***			***x***	***x***					***5***
***Process—definition according to CFIRGuide*** [84]																	
Planning—the degree to which a scheme or method of behavior and tasks for implementing an intervention are developed in advance, and the quality of those schemes or methods											***x***						**1**
*Engaging*—*attracting and involving appropriate individuals in the implementation and use of the intervention through a combined strategy of social marketing, education, role modeling, training, and other similar activities*			***x***		***x***			***x***			***x***			***x***			**5**
*Formally appointed internal implementation leaders*—*individuals within the organization who have been formally appointed to be responsible for implementing an intervention as the coordinator, project manager, team leader, or other similar role*							***x***					***x***					**2**
*Champions*—*individuals who dedicate themselves to supporting, marketing, and driving through an implementation, while overcoming indifference or resistance that the intervention may provoke in an organization*			***x***	***x***	***x***		***x***	***x***			***x***			***x***			**7**
*External change agents*—*individuals who are affiliated with an outside entity who formally influence or facilitate intervention decisions in a desirable direction*								***x***									**1**
Reflecting and evaluating—quantitative and qualitative feedback about the progress and quality of implementation accompanied by regular personal and team debriefing about progress and experience				***x***			***x***	***x***									**3**

#### Intervention characteristics

The *adaptability* of the intervention was the most frequently reported CFIR construct within this domain. The *adaptability* of the intervention was described in terms of the needs of people with dementia and their relatives [61, 64, 66, 70, 71], knowledge that is needed/required [62, 78] and interests of professionals [64, 78], the user-friendliness of the intervention [66], organizational interests [62], and resources such as time [62, 69, 78] and staffing [64], as well as local sites where it would be interesting to implement the intervention [81].

*Evidence strength and quality* of the intervention was described as the second most common CFIR construct (Table 6) and was reported in terms of the perceived *evidence strength and quality* of the intervention [60, 63, 64] or related to intervention components such as the specialized staff (e.g., ANPs) and their roles, competencies, and skills [70, 71, 82]; information materials; documents [70, 71]; tools [77]; trainings [63, 77]; the environment [71]; and procedures [71].

#### Outer setting

We identified *patient needs and resources* as the most reported CFIR construct in this domain. Due to the focus on people with dementia and the importance of relatives as proxies during the care process, we additionally included aspects such as the *needs and resources of families* (which are not included in the original CFIR). *Patient needs and resources* were primarily described in relation to dementia [70] and were understood as influencing factors that impact implementation outcomes. For example, learning ability and the ability to coordinate care, the perception of the acute disease regarding severity and the implication of their symptoms [70] were described as influencing factors. In addition, intervention fidelity [70, 82], attitudes toward the intervention [70], and the ability to use the intervention and the awareness of the staff to support the use of the intervention [61], as well as patient resources (such as finances, living environment, insurance and medication coverage, access to healthcare, and the social network), were reported as influencing factors [70].

Influencing factors regarding *needs and resources of the family* were reported in terms of caregiver burden [70, 71, 82], skills and knowledge of the family (caregiver) related to the care [61, 70, 77, 82], and its coordination [70] as well as the knowledge about [70] and the perception of the disease (acute disease and dementia) [70]. In addition, expectations [61] and acceptance of the intervention [70], information about and participation in the intervention and its design [61, 64, 70, 77] were also described as influencing factors regarding the family.

*Cosmopolitanism* was described as the second most common construct in this domain. Here, the support and involvement of external networks such as the Alzheimer’s Association was described as an influencing factor on implementation [67, 82]. The fragmentation of the healthcare system and therefore the provision of care services was also reported as an influencing factor in the studies. In this context, aspects such as lack of cooperation, shared care plans and information exchange between external actors (e.g., primary care physicians, specialist clinics) were mentioned [71, 82].

#### Inner setting

*Structural characteristics*, *culture*, and *networks and communications* were identified as the most mentioned CFIR constructs in this domain. For the constructs *implementation climate* and *readiness for implementation*, the subcodes with the most frequent descriptions were *learning climate* and *available resources*.

Reported influencing factors within the *structural characteristics construct* were staff turnover [59, 67, 72, 77], structural changes in medical specialization [72], the physical environment [79], the work organization (e.g., shift work, double shifts and high volume of agency staff) [69, 77], and the level of awareness of cognitive impairment (dementia and delirium) [67]. The care setting itself was mentioned as a general influencing factor with an impact on the implementation [69, 77].

The construct *culture* was described as an influencing factor in terms of the culture of the organization in general [79] and management style [61, 64].

The construct *networks and communications* included exchange options such as meetings [62, 71], interdisciplinary teamwork [71, 72, 81], and time points when these options were available [71] during the implementation process as influencing factors.

Within the construct *implementation climate*, learning climate was the most described subcode, including influencing factors related to space for learning (for example, mentoring or supervision [61, 77]), as well as involvement [61, 77], support [72, 77, 81], and acknowledgment [61] of the staff during the implementation process, opportunities to try out new methods [70], and feeling safe [61] while using the intervention even if others (e.g., relatives or colleagues) disagree.

Reported influencing factors within the construct *readiness for implementation* were more often related to the subcode *available resources*, which includes time and workload of the staff [59–63, 67, 69, 72, 77–79, 81], staffing level [62, 71, 77], and resources for training [61]. Additionally, the physical environment, such as walking areas and activity rooms [77], and activity materials [77] and finances of the facility were mentioned [77].

#### Characteristics of individuals

We identified *knowledge and beliefs about the intervention* and *other personal attributes* as the most mentioned constructs for this CFIR domain.

*Knowledge and beliefs about the intervention* were described by the influencing factor attitude toward the intervention, for example acceptance [60, 61, 77], usefulness [60, 63, 71, 72, 77, 81], appropriateness [63, 71, 77], agreement with values [63, 72], burden [77], and extra work [77]. Moreover, the expectation of the intervention (e.g., outcomes) or its implementation (e.g., losing jobs) [64] was also described and included reports about desired or perceived outcomes for the patient and the family (e.g., well-being, quality of life, relationship, positive response) [61, 64, 77, 81], the staff (e.g., empowerment, confidence, teamwork, work satisfaction) [61, 64, 77], and the organization (e.g., reputation, public relations, requesting new entries, time, and workload) [61, 71]. Furthermore, the knowledge about the intervention and their task and roles in providing these interventions were described as additional influencing factors [61, 63, 71, 72].

Influencing factors such as motivation [64, 77], commitment [61, 69], language [61], experience [67], social skills [79], openness [62, 77], and cooperativeness [61] were identified as *other personal attributes*.

#### Process

In this CFIR construct, we identified the most frequently influencing factors related to *engaging*. We found influencing factors on engaging in general as well as specific influencing factors related to champions.

*Engaging* was reported in terms of engagement of staff in general (e.g., existing or lack of) [59, 72, 79, 81], qualities of the people engaged (e.g., interdisciplinarity and skills in dementia care) [62, 67, 77], and strategies (e.g., relocation staff members) [77].

Influencing factors related to champions were distinguished in quality (e.g., strong and passionate about dementia care, expertise in dementia care, skills and interest in the intervention) [62, 72, 77], tasks (e.g., interdisciplinary problem solving, ongoing education, brainstorming activities, staff meetings, physical presence on the ward) [59, 61, 64, 72, 77], and roles (e.g., role modeling, leading light in the implementation process) [59, 61, 77]. Moreover, the availability of a champion was reported as a general influencing factor [59, 67, 72].

## Discussion

To our knowledge, this is the first study to systematically identify implementation strategies, implementation outcomes and influencing factors related to the implementation of evidence-based interventions that focus on three preselected phenomena in people with symptoms of dementia or those who have been diagnosed with dementia: (A) behavior that challenges supporting a person with dementia in long-term care, (B) delirium in acute care, and (C) postacute care needs. The strengths of our scoping review are the methodological quality and the systematic and broad scope. Consequently, we can provide a broad and theoretically guided overview of the current state of implementation research in dementia care across different healthcare settings.

In summary, we identified various multifaceted implementation strategies (between 4 and 21 per study), implementation outcomes (between 0 and 5 per study), and influencing factors (between 1 and 19 per study) across the 13 included studies [59–67, 69–72]. Despite the three different dementia-specific phenomena and the different healthcare settings, we did not find remarkable differences in the use of the implementation strategies, implementation outcomes, or factors influencing the implementation.

In terms of influencing factors, *available resources* appeared to be one of the most important factors influencing implementation, along with the *adaptability* of the intervention. This does not come as a surprise since acute care and nursing homes have often struggled with staffing, high staff turnover rates, funding issues, challenges with available equipment, and limited influence on changing the environment, even before the COVID-19 pandemic [85–88]. This could explain why we found hardly any differences in the reported implementation strategies and influencing factors between the different interventions and settings. Accordingly, it appears that these contextual factors tremendously influence the successful implementation of evidence-based interventions due to their general conditions and requirements for implementation under current conditions (e.g., staffing, staff workload, competencies, qualifications, turnover, finances). These current contextual factors can be understood as an implementation-hostile climate [89]. To address this challenge, the implementability of healthcare interventions seems to be a crucial point [90], and adapting the intervention to the specific care context and professionals’ workflows for higher acceptability will be key for successful implementation [91]. This highlights the importance of not developing and evaluating interventions in isolation from implementation strategies [92, 93] and/or without a process evaluation [94–96].

Furthermore, it seems necessary to critically discuss the added value of implementation research with a sole focus on influencing factors, even when this could lead to the identification of defining implementation strategies [38]. Here, a paradigm shift [97] from identifying and describing these influencing factors to developing concrete evidence-based implementation strategies seems necessary. Thus, for the discipline (implementation science) to move forward, it is essential to consolidate innovative study designs [98] and methods (specifically participatory research approaches [99]) to develop discrete, multifaceted, and tailored implementation strategies and to investigate/test their impact on the implementation strategy and outcome itself as well as the effect on intervention outcomes [100]. This gap in the current implementation research is confirmed by our results since we were only able to identify one study that tested the effectiveness of an implementation strategy [65]. Consequently, the effects of the implementation strategies we identified are still largely unknown, and it seems that implementation research [101] and respective process evaluations to address implementation challenges during the evaluation of an intervention [93] in dementia care have barely evolved in relation to this point.

However, there also seems to be a lack in the reporting of implementation outcomes and the use of psychometrically tested implementation outcome measurements, as well as an inconsistency in the understanding between intervention outcomes and implementation outcomes [47, 102, 103]. For example, in our included studies, implementation outcomes were often not specifically named as such and were not measured with psychometric tested assessments, or it often remained unclear to what extent the measurement of, e.g., gaining knowledge, could be either an implementation outcome or an outcome of an intervention if the focus lies on education. Therefore, it is necessary to improve reporting on implementation strategies and outcomes (in both intervention and implementation studies) to initiate the development of psychometrically tested measurements [102] and, despite the publication of Proctor, Silmere [37] in 2011, to keep in mind the tension between intervention and implementation outcomes [47].

Finally, we were able to identify dementia-specific influencing factors, in particular related to the family, their needs and resources, as a key point during the implementation of evidence-based interventions. This meant that we needed to modify the CFIR (outer setting*—patient needs and resources/needs and resources of the family*) for our review accordingly. Although the updated version of the CFIR was published in 2022 [104], considering family needs and resources as an influencing factor for implementation does not seem to be included. However, from our perspective, this seems to be a highly relevant factor for older people with and without dementia [105]. In addition, other dementia-specific influencing factors also appear to exist for the implementation of interventions that include this population [106]. We live in a diverse and global world, and in the health sector, embracing diversity is essential for individuals’ health [107, 108]. Here, it seems to be of interest in future (implementation) research to what extent frameworks such as the CFIR consider factors influencing diverse populations (e.g., people with dementia and/or migrants or ethnic minority groups). In summary, these aspects could lead to further and tailored development of the CFIR as well as the ERIC.

## Limitations

Our scoping review has some limitations. As a first step, we derived our search terms from identified exemplary evidence-based dementia care interventions and their components (e.g., DICE) and supplemented them with other, broader terms (e.g., person-centered care). In doing so, we cannot exclude the possibility that we failed to consider very specific interventions addressing our preselected dementia phenomena. However, across the different included studies and thus the different interventions and settings, our results present a very homogeneous picture regarding influencing factors, implementation strategies, and outcomes. Second, by using the ERIC clusters, Proctor’s outcomes, and the CFIR domains and constructs, we used specific frameworks and descriptions, which makes it difficult to compare our results with others analyzed with other frameworks and descriptions. However, the ones we used are among the most established due to their high number of citations [57, 109]. Third, we need to point out that an update of the CFIR [104] and the CFIR Outcomes Addendum [110] were published after the completion of our review (2021). In particular, the update of the CFIR is characterized by a more specific and detailed classification of the different influencing factors (e.g., subdividing patient needs and resources into three different constructs and moving them into the domain of internal setting and persons). Therefore, it would be interesting to compare our results with the results of future dementia-specific studies focusing on influencing factors and using the updated CFIR. It would be interesting to analyze the extent to which the updated CFIR is in line with our understanding of influencing factors. Damschroder, Reardon [104] point out that despite the changes in the updated CFIR version, the constructs can be consistently mapped back to the original CFIR, thus allowing comparison of their conceptualization.

Finally, it should be mentioned that publication bias cannot be excluded; for example, we did not specifically and systematically search for gray literature [111].

## Conclusion

Based on the ERIC, the descriptions of Proctor, Silmere [37], and the CFIR, our scoping review provides a broad but systematically conducted and structured overview of the current state of implementation research in dementia care. Furthermore, our review identifies various gaps to be addressed by further implementation research. Our results show that the factors influencing the implementation of evidence-based interventions in dementia care are highly homogeneous, regardless of the evidence-based intervention and/or healthcare setting. In addition, the influencing factors we identified most frequently (available resources and adaptability of the intervention) are factors to be expected in the context of and with an impact on the provision of dementia care. In contrast, we found almost no reports on the effects of the identified implementation strategies. Consequently, to fill this gap, it seems important to test existing implementation strategies, to address tailoring-based awareness for the known influencing factors and to advance implementation science and therefore to be able to make predictions about the effectiveness of implementation strategies. This could further promote the overall translation of evidence-based dementia care practice and sustain a high quality of care for a vulnerable population.

### Supplementary Information

**Additional file 1: Supplementary Table 1.** Preferred Reporting Items for Systematic reviews and Meta-Analyses extension for Scoping Reviews (PRISMA-ScR) checklist [24]. **Supplementary Table 2.** Search strategy example in MEDLINE for behavior that challenges supporting a person with dementia in long-term care (via PubMed). **Supplementary Table 3.** Search strategy example in MEDLINE for delirium (via PubMed). **Supplementary Table 4.** Search strategy example in MEDLINE for postacute care needs (via PubMed).

## Data Availability

All data generated or analyzed during this study are included in this published article and its supplementary information files.
